# *Plasmodium falciparum* In Vitro Resistance to Monodesethylamodiaquine, Dakar, Senegal, 2014

**DOI:** 10.3201/eid2205.151321

**Published:** 2016-05

**Authors:** Bécaye Fall, Marylin Madamet, Cheikhou Camara, Rémy Amalvict, Mansour Fall, Aminata Nakoulima, Bakary Diatta, Yaya Diémé, Boubacar Wade, Bruno Pradines

**Affiliations:** Hôpital Principal de Dakar, Dakar, Senegal (B. Fall, C. Camara, M. Fall, A. Nakoulima, B. Diatta, Y. Diémé, B. Wade, B. Pradines);; Institut de Recherche Biomédicale des Armées, Brétigny sur Orge, France (M. Madamet, R. Amalvict, B. Pradines);; Aix Marseille Université, Marseille, France (M. Madamet, R. Amalvict, B. Pradines);; Centre National de Référence du Paludisme, Marseille, France (M. Madamet, R. Amalvict, B. Pradines)

**Keywords:** Plasmodium falciparum, malaria, antimalarial drug, in vitro, parasites, resistance, Dakar, Senegal, vector-borne infections, antimicrobial resistance

## Abstract

We successfully cultured 36 *Plasmodium falciparum* isolates from blood samples of 44 malaria patients admitted to the Hôpital Principal de Dakar (Dakar, Senegal) during August–December 2014. The prevalence of isolates with in vitro reduced susceptibility was 30.6% for monodesethylamodiaquine, 52.8% for chloroquine, 44.1% for mefloquine, 16.7% for doxycycline, 11.8% for piperaquine, 8.3% for artesunate, 5.9% for pyronaridine, 2.8% for quinine and dihydroartemisinin, and 0.0% for lumefantrine. The prevalence of isolates with reduced in vitro susceptibility to the artemisinin-based combination therapy partner monodesethylamodiaquine increased from 5.6% in 2013 to 30.6% in 2014. Because of the increased prevalence of *P. falciparum* parasites with impaired in vitro susceptibility to monodesethylamodiaquine, the implementation of in vitro and in vivo surveillance of all artemisinin-based combination therapy partners is warranted.

## Abstract 2

In vitro and in vivo surveillance of all artemisinin-based combination therapy partners is warranted.

In 2004, Senegal adopted the use of sulfadoxine/pyrimethamine with amodiaquine as the first-line therapy for malaria in response to increasing chloroquine resistance. In 2006, the National Malaria Control Program of Senegal recommended artemisinin-based combination therapy (ACT) as the first-line treatment for uncomplicated malaria (*1*,*2*). The combined sulfadoxine/pyrimethamine and amodiaquine treatment was then changed to artemether/lumefantrine or artesunate/amodiaquine. The number of reports assessing levels of *Plasmodium falciparum* resistance to antimalarial drugs since the introduction of ACT in Senegal has been limited. Changes in resistance to antimalarial drugs were observed during 2008–2011 in Thiès, the third largest city in Senegal, when parasites became less susceptible to amodiaquine, artemisinin, and chloroquine (*3*). The ex vivo susceptibility to monodesethylamodiaquine, which is the active metabolite of amodiaquine, has been low and stable for the past 10 years in Dakar (6.0% in 2009, 11.8% in 2010, and 5.6% in 2013) (*4*–*6*). The prevalence of reduced susceptibility to lumefantrine remains <3.0% (*4*–*6*). To determine whether parasite susceptibility has been affected by the use of ACT, we conducted an ex vivo susceptibility study on local isolates from Dakar obtained from the Hôpital Principal de Dakar during August–December 2014. The malaria isolates were assessed for susceptibility to standard drugs, such as monodesethylamodiaquine (the active metabolite of amodiaquine), lumefantrine, chloroquine, quinine, mefloquine, artesunate, dihydroartemisinin (the active metabolite of artemisinin derivatives), doxycycline, and new antimalarial drugs (e.g., pyronaridine and piperaquine).

## Materials and Methods

### Patients and Sample Collection

We obtained blood samples 44 *P. falciparum* malaria patients admitted to the Hôpital Principal de Dakar (Dakar, Senegal) during August–December 2014. Of the 44 patients, 73% were recruited from the emergency department; other patients were recruited from the intensive care unit (7%), pediatric department (13%), or other units (9%). Venous blood samples were collected from each patient by using Vacutainer acid citrate dextrose tubes (Becton Dickinson, Rutherford, NJ, USA) before treatment began. Informed verbal consent from the patients or their parents/guardians was obtained before blood collection. This study was approved by the ethics committee of the Hôpital Principal de Dakar. 

For all 44 patients, no information was available on antimalarial treatment before admission. Previous intake of antimalarial drugs can affect the phenotype of parasites isolated from patients. Despite World Health Organization recommendations, the patients were treated with quinine until November 2014, and then with artesunate or artemether/lumefantrine. 

Thin blood smears were stained using a RAL kit (Réactifs RAL, Paris, France) by using eosin and methylene blue and were examined to determine *P. falciparum* density and confirm monoinfection. The level of parasitemia ranged from 0.13% to 14.13% for male patients (n = 31) and from 0.06 to 11.61% (n = 13) for female patients.

Parasitized erythrocytes were washed 3 times in RPMI 1640 medium (Invitrogen, Paisley, UK) buffered with 25 mmol/L HEPES and 25 mmol/L NaHCO_3_. If parasitemia exceeded 0.1%, infected erythrocytes were diluted to 0.1% with uninfected erythrocytes (human blood type A+) and resuspended in RPMI 1640 medium supplemented with 10% human serum (Abcys S.A., Paris, France), for a final hematocrit of 1.5%. The susceptibility of the isolates was assessed without culture adaptation.

### Drugs

Chloroquine, quinine, doxycycline, and dihydroartemisinin were purchased from Sigma-Aldrich (Saint Louis, MO, USA). Monodesethylamodiaquine was obtained from the World Health Organization (Geneva, Switzerland). Mefloquine was purchased from Roche (Paris, France), and lumefantrine was purchased from Novartis Pharma (Basel, Switzerland). Artesunate, piperaquine, and pyronaridine were obtained from Shin Poong Pharm Co. (Seoul, Korea). 

Quinine, monodesethylamodiaquine, mefloquine, dihydroartemisinin, artesunate, piperaquine, and doxycycline were dissolved in methanol and then diluted in water to final concentrations ranging from 6 nmol/L to 3,149 nmol/L for quinine, 1.9 nmol/L to 1,988 nmol/L for monodesethylamodiaquine, 1.5 nmol/L to 392 nmol/L for mefloquine, 0.1 nmol/L to 107 nmol/L for dihydroartemisinin, 0.1 nmol/L to 98 nmol/L for artesunate, 1.9 nmol/L to 998 nmol/L for piperaquine and 0.1 μmol/L to 502 µmol/L for doxycycline. Chloroquine and pyronaridine were resuspended and diluted in water to final concentrations ranging from 6 nmol/L to 3,231 nmol/L and 0.4 nmol/L to 199 nmol/L, respectively. Lumefantrine was resuspended and diluted in ethanol to obtain final concentrations ranging from 0.6 nmol/L to 310 nmol/L.

We tested and validated the batches of plates on the chloroquine-resistant W2 strain (Indochina) (Malaria Research and Reference Reagent Resource Center, Manassas, VA, USA) in 5 independent experiments. The clonality of the W2 strain was verified every 15 days by using PCR genotyping of the polymorphic genetic markers *msp1* and *msp2* and microsatellite loci (*7*,*8*) and annually by an independent laboratory from the Worldwide Antimalarial Resistance Network.

### Ex Vivo Assay

For the in vitro microtests, we aliquoted 100 µL of parasitized red blood cells (final parasitemia 0.1%, final hematocrit 1.5%) into 96-well plates predosed with antimalarial drugs (monodesethylamodiaquine, lumefantrine, chloroquine, quinine, mefloquine, dihydroartemisinin, artesunate, piperaquine, pyronaridine, and doxycycline). The plates were incubated in a sealed bag for 72 hours at 37°C with atmospheric generators for capnophilic bacteria by using Genbag CO_2_ at 5% CO_2_ and 15% O_2_ (BioMérieux, Marcy l’Etoile, France) (*9*).

After thawing the plates, we homogenized the hemolyzed cultures by vortexing the plates. The success of the drug susceptibility assay and the appropriate volume of hemolyzed culture to use for each assay were determined for each clinical isolate during a preliminary histidine-rich protein 2 ELISA. Both the pretest and subsequent ELISA tests were performed using a commercial kit (Malaria Ag Celisa, Cellabs PTY LTD, Brookvale, Australia) in accordance with the manufacturer’s recommendations. The optical density (OD) of each sample was measured with a spectrophotometer (Multiskan EX, Thermo Scientific, Vantaa, Finland).

The 50% inhibitory concentration (IC_50_) for each of the 10 drugs was calculated with the inhibitory sigmoid E_max_ model, which estimated the IC_50_ through nonlinear regression by using a standard function of the R software (ICEstimator version 1.2, http://www.antimalarial-icestimator.net) (*10*). IC_50_ values were validated only if the OD ratio (OD at zero concentration / OD at maximum concentration) was >1.6 and the 95% CI of the IC_50_ estimation was <2.0 (*10*). The cutoff values for in vitro resistance or reduced susceptibility were as follows: 77 nmol/L for chloroquine, 61 nmol/L for monodesethylamodiaquine, 115 nmol/L for lumefantrine, 12 nmol/L for dihydroartemisinin, 12 nmol/L for artesunate, 611 nmol/L for quinine, 30 nmol/L for mefloquine, 135 nmol/L for piperaquine, 60 nmol/L for pyronaridine, and 37 µmol/L for doxycycline (*4*,*11*). 

### Data and Statistical Analysis

IC_50_ values were analyzed after logarithmic transformation. Values were expressed as the geometric mean of the IC_50_ with 95% CI.

## Results

From the blood samples collected from the 44 *P. falciparum* malaria patients admitted to the Hôpital Principal de Dakar during August–December 2014, we successfully cultured a total of 36 isolates, and calculated the average parameter estimates for the 10 antimalarial drugs (Table). Only 44 *P. falciparum* malaria cases were reported in Dakar during the 4-month study period; a 27.6% decrease in malaria prevalence occurred in Senegal during 2013–2014 (*12*).

**Table:** Ex vivo susceptibility to standard antimalarial drugs of 36 *Plasmodium falciparum* isolates from 44 malaria patients compared with a *P. falciparum* W2 clone tested under the same conditions, Hôpital Principal de Dakar, Dakar, Senegal, August 2014–December 2014*

Antimalarial drug	Geometric mean IC_50_ (95% CI)†		Ratio of geometric mean IC_50_ (isolate/W2)	Cutoff for reduced susceptibility†	% Isolates with reduced susceptibility (no./no. tested)
	Isolates	W2 clone			
Monodesethylamodiaquine	25.3 (16.9–38.0)	70 (66–74)	0.36	61	30.6 (11/36)
Lumefantrine	6.8 (4.4–10.8)	15.4 (11.7–20.3)	0.44	115	0 (0/36)
Chloroquine	64.6 (46.2–90.2)	254 (234–276)	0.25	77	52.8 (19/36)
Mefloquine	22.6 (16.9–30.3)	12.7 (11.5–14.1)	1.78	30	44.1 (15/34)
Quinine	80.2 (54.4–118.2)	262 (247–278)	0.31	611	2.8 (1/36)
Piperaquine	36.4 (26.2–50.6)	34.8 (31.9–37.9)	1.05	135	11.8 (4/34)
Pyronaridine	10.5 (7.8–14.1)	26.0 (23.9–28.3)	0.40	60	5.9 (2/34)
Dihydroartemisinin	1.8 (1.17–2.77)	1.26 (1.05–1.57)	1.43	12	2.8 (1/36)
Artesunate	2.5 (1.6–3.7)	1.19 (1.03–1.41)	2.10	12	8.3 (3/36)
Doxycycline	8.5 (5.6–12.7)	10.4 (9.2–11.7)	0.82	37	16.7 (6/33)

*The geometric mean IC_50_ values for W2 are the results of 5 independent experiments, in which batches of plates were tested and validated on the chloroquine-resistant W2 strain (Indochina). IC_50_, 50% inhibitory concentration. †All IC_50_ values are given in nmol/L except those for doxycycline, which are given in µmol/L.

The prevalence of isolates with in vitro reduced susceptibility was 30.6% for monodesethylamodiaquine, 52.8% for chloroquine, 44.1% for mefloquine, 16.7% for doxycycline, 11.8% for piperaquine, 8.3.0% for artesunate, 5.9% for pyronaridine, 2.8% for quinine and dihydroartemisinin, and 0% for lumefantrine. The prevalence of isolates with in vitro reduced susceptibility to monodesethylamodiaquine increased significantly, from 5.6% in 2013 (*6*) to 30.6% in 2014 (p = 0.04 by Pearson χ^2^ test). Six isolates had high monodesethylamodiaquine IC_50_, defined as >100 nmol/L (101 nmol/L, 108 nmol/L, 140 nmol/L, 158 nmol/L, 161 nmol/L, and 227 nmol/L).

## Discussion

Longitudinal in vitro analysis of the susceptibility of *P. falciparum* isolates to antimalarial drugs has 3 benefits (*13*). First, this approach enables assessment of the response of clinical isolates to individual drugs regardless of host factors that influence drug efficacy in vivo. The response of a patient to drug treatment is complex and reflects host factors and intrinsic responses of the parasite to the drug. This approach enables surveillance for resistance to both components of a drug combination such as ACT. In vitro testing is the only method that is currently available to provide clear early warning of impending resistance to the components of ACT. Second, tracking the progressive decline in drug susceptibility from the same site is likely to be the most sensitive method to identify growing resistance in a parasite population. Third, strains with reduced susceptibilities can be established in continuous culture as stable reference lines to provide the tools needed to investigate novel molecular mechanisms and to define the baseline of susceptibility to a new drug.

In vitro analysis of cross-resistance among drugs is crucial to avoid development or introduction of new drugs to which parasites are already resistant. It is also important to use the molecules that actually act in humans (i.e., the antimalarial drugs themselves) if they act directly without metabolization or with their active metabolites (e.g., dihydroartemisinin for artemisinin derivatives or monodesethylamodiaquine for amodiaquine).

In the absence of standardized ex vivo and in vitro tests, it is very difficult to compare data from different laboratories’ IC_50_ and cutoff values for in vitro resistance are specific to the methodology. The in vitro effects and the IC_50_ values for antimalarial drugs depend on incubation conditions (*14*,*15*), gas conditions (e.g., the effects of O_2_ and CO_2_) (*9*,*16*), and methodology (e.g., use of an isotopic test vs. an immunoenzymatic test) (*17*). These differences in methodology must be taken into account when comparing and analyzing resistance data from different studies.

Our methodology was the same as that used during 2013–2014 (*6*), which enables comparison of the data. In addition, the W2 clone was used as an internal control in both studies. Comparison of W2 susceptibility data for the 10 antimalarial drugs in 2014 to those of previous years indicated no significant difference between the 2 studies in terms of response to antimalarial drugs (0.45<p<0.91).

The prevalence of isolates with in vitro reduced susceptibility to monodesethylamodiaquine increased significantly from 5.6% in 2013 (*6*) to 30.6% in 2014 (p = 0.04). In the absence of a significant difference in W2 responses to monodesethylamodiaquine between the 2 studies, the increase in the IC_50_ geometric mean and the prevalence of reduced in vitro susceptibility are attributable to the evolution of monodesethylamodiaquine susceptibility and not differences in methodology. There are 2 hypotheses that might explain the observed increase: 1) the use of artesunate/amodiaquine in Senegal generated the emergence of amodiaquine-resistant parasites (*1*,*2*), or 2) cross-resistance has occurred between chloroquine and monodesethylamodiaquine (*4*,*18*). A decrease in chloroquine resistance that was observed in Dakar during 2009–2011 parallels the withdrawal of chloroquine treatment (*4*,*5*). However, the prevalence of in vitro resistance to chloroquine increased again in Dakar to 50% in 2013 (*6*) and 52.8% in 2014. This phenomenon had already been observed in the Dakar suburb of Pikine, where malaria parasites demonstrated an increase in the *pfcrt* 76T mutation involved in chloroquine resistance (*19*). During 2011–2012, the efficacy of artesunate/amodiaquine was 99.3% in Senegal (*20*).

The other ACT first-line treatment for uncomplicated *P. falciparum* malaria in Senegal is the combination of artemether and lumefantrine. No isolates with reduced susceptibility to lumefantrine have been detected, and prevalence of isolates with reduced susceptibility to lumefantrine has remained <3% in Dakar since the introduction of ACT (*4*–*6*). During 2011–2012, the efficacy of artemether/lumefantrine was 100% in Senegal (*20*). At the Hôpital Principal de Dakar, the patients from this study were treated with quinine until November 2014. The patients were then treated with artesunate or artemether/lumefantrine. All 44 of the patients fully recovered.

A new ACT second-line treatment for uncomplicated *P. falciparum* malaria is the combination of dihydroartemisinin and piperaquine. During 2011–2012, the efficacy of dihydroartemisinin/piperaquine was 100% in Senegal (*20*). The geometric mean IC_50_ values for piperaquine (34.8 nmol/L) observed in Dakar in 2014 were comparable to those observed in 2013 (32.2 nmol/L) (*6*). The prevalence of isolates with reduced susceptibility to piperaquine was 11.8% in 2014 in Dakar.

The pyronaridine/artesunate combination is one of the most recent ACT combinations to be considered and is currently under development. The prevalence of isolates with reduced susceptibility to pyronaridine was 5.9% in 2014 in Dakar. The geometric mean IC_50_ values for pyronaridine (10.5 nmol/L) observed in Dakar in 2014 were higher than those observed in Dakar in 2013 (5.8 nmol/L) (*6*) or in Dielmo in 1996 and 1997 (3.8 nmol/L and 4.52 nmol/L) (*21*,*22*).

The present study showed that 2.8% and 8.3% of the isolates in 2014 were less susceptible to dihydroartemisinin and artesunate, respectively. Previous studies found that no parasites were resistant to these 2 drugs in Dakar (*4*–*6*,*23*). However, the standard in vitro test was not adapted to follow resistance to artemisinin derivatives. The clinical resistance to artemisinin was manifested by an increase in the ring-stage survival rate after contact with artemisinin (*24*).

The prevalence of isolates with reduced susceptibility to mefloquine remained high in Dakar in 2014 (44.1%) compared with prevalences observed in 2001 (17%) and 2002 and (13%) (*23*,*25*) but was relatively stable compared with 2009 (50%–62%) (*4*–*6*). Clinical trials are in progress to evaluate the efficacy of mefloquine for intermittent preventive treatment of infants and pregnant women with *P. falciparum* malaria. Nevertheless, mefloquine has been used relatively infrequently in Africa compared with Asia.

In 2014, 2.8% of the isolates showed low reduced susceptibility to quinine. This finding is consistent with those of previous studies conducted in Dakar (*4*–*6*,*23*,*25*). All of the patients treated with quinine until November 2014 fully recovered.

The prevalence of parasites with reduced susceptibility to doxycycline increased slightly in 2014 (16.7%) compared with previous years (10.3%–12.0%). However, the geometric mean IC_50_ was lower (8.5 µmol/L for 2014 vs. 9.2 µmol/L for 2010 and 11.6 µmol/L for 2009) (*4*,*5*).

Because of the short half-life of artemisinin derivatives, they have been paired with longer-lived partners, such as amodiaquine, lumefantrine, or piperaquine, for longer drug action and prophylaxis against reinfecting parasites. Because of the increased prevalence of *P. falciparum* parasites with impaired in vitro susceptibility to monodesethylamodiaquine in Dakar in 2014, implementation of in vitro and in vivo surveillance of all ACT partners is warranted. This in vitro reduced susceptibility to monodesethylamodiaquine might soon affect the in vivo efficacy of artesunate/amodiaquine, which will become the equivalent of a monotherapy using only artesunate if this in vitro resistance is confirmed in the future, especially because resistance to artesunate has already emerged in Asia (e.g., Cambodia, Thailand, Myanmar, Vietnam, China, and India) (*26*). In addition, in Senegal, it will be a priority to identify mutations in the *PF3D7_1343700* kelch propeller domain. In southern Asia, these mutations have been associated with in vitro resistance to artemisinin and delayed clearance after artemisinin treatment (*27*).
